# Rare case of pulmonary fat embolism and acute respiratory distress syndrome after liposuction and fat grafting: a case report

**DOI:** 10.3389/fmed.2023.1202709

**Published:** 2023-05-23

**Authors:** Xiaoyan Gai, Xiaoyan Sun, Xiang Zhu, Qingtao Zhou, Yongchang Sun

**Affiliations:** ^1^Department of Respiratory and Critical Care Medicine, University Third Hospital, Beijing, China; ^2^Department of Pathology, Peking University Third Hospital, Beijing, China

**Keywords:** acute respiratory distress syndrome, fat grafting, liposuction, pulmonary fat embolism, bronchoalveolar lavage (BAL)

## Abstract

**Background:**

Pulmonary fat embolism usually occurs after fracture, yet rarely observed after liposuction and fat grafting.

**Case presentation:**

We describe a 19-year-old female patient who presented with acute respiratory failure and diffuse pulmonary opacities on chest radiographic image shortly after liposuction and fat grafting. Bronchoalveolar lavage was performed and lipid content in alveolar cells contribute to the diagnosis of the fat embolism syndrome. The patient was successfully treated with noninvasive mechanical ventilation and a short course of glucocorticoids.

**Conclusions:**

Early recognition and appropriate treatment are very important to improve the outcome of pulmonary fat embolism. Considering that liposuction and fat grafting are increasingly common cosmetic surgeries, our aim is to raise awareness for this rare adverse event.

## Background

Fat embolism syndrome (FES) often occurs after trauma, such as long bone fractures (1). Fat embolism following liposuction is a rare complication with few cases reported worldwide (2). The common clinical manifestations of FES include lung, central nervous system, and skin symptoms, which usually occur 12–72 h after trauma. The most typical triple sign is hypoxemia, decreased level of consciousness, and petechial rash. There is no standardized validated diagnostic test for FES. The diagnosis is challenging and often achieved in most cases based on clinical manifestations, imaging, and risk factors. We report a patient presenting with acute hypoxemia, respiratory distress, and typical blizzard-like changes on chest computed tomography (CT) hours after liposuction.

## Case presentation

A 19-year-old woman was admitted to the emergency department with chest tightness persisting for 2 h. She had a six-year history of unhealthy habits including fasting and self-induced vomiting to control weight and was diagnosed with an eating disorder in a psychiatric hospital. In the past 2 years, she had undergone zygomatic plastic surgery with subsequent rhinoplasty. Ten h before presentation, she underwent liposuction and fat grafting (liposuction of the limbs and hips and fat grafting in the face and chest), lasting for 3 h under general anesthesia. Approximately 8 h after the operation, she experienced chest tightness and palpitations in the ward, with a heart rate of 120 beats/min, and her peripheral blood oxygen saturation decreased to 80% on room air. She had a fever of 37.4°C and coughed up a small amount of yellow phlegm. No shivering, headache, dizziness, systemic rash and other clinical signs were noted. She was immediately treated with non-invasive mechanical ventilation and transferred to the Respiratory Intensive Care Unit (RICU).

Vital signs at the entrance of RICU revealed a temperature of 37.2°C, blood pressure of 97/62 mmHg, heart rate of 120 beats/min, respiratory rate of 18 breaths/min, and blood oxygen saturation level of 96% (non-invasive mechanical ventilation at FiO_2_ of 50%). Height of 162 cm, weight of 48 kg. The whole body was wrapped in compression bandages. Breathing sounds in both lungs were decreased, with no wheezing, crackles, or rhonchi. Cardiovascular examination revealed normal heart sounds without murmur or rubs. There was no swelling of the upper limbs; both lower limbs were wrapped in elastic bandages and exhibited mild tenderness.

Complete blood examination revealed the following: white blood cell count, 10.52 × 10^9^/L; neutrophils, 88.9%, hemoglobin, 99 g/L; rapid C-reactive protein level, 11.9 mg/L; serum procalcitonin, 0.08 ng/mL; albumin, 22.2 g/L; total cholesterol, 0.03 mmol/L; triglyceride, 0.01 mmol/L; high-density lipoprotein cholesterol, 0.02 mmol/L; low-density lipoprotein cholesterol, 0.02 mmol/L; D-dimer, 0.28 μg/mL; and brain natriuretic peptide, 1,104 pg/mL. Liver and renal functions and cardiac enzyme and troponin T levels were normal. Arterial blood gas analysis (on room air) showed a pH of 7.32, PaCO_2_ of 31 mmHg, PaO_2_ of 53 mmHg, HCO3- of 15.90 mmol/L, and lactic acid level of 2.37 mmol/L. Arterial blood gases on admission (under 5 cm H_2_O of positive end-expiratory pressure and FiO_2_ 45%) showed a pH of 7.39, PaO_2_ of 76 mmHg, with a PaO_2_/FiO_2_ ratio of 168.

**Figure 1:**
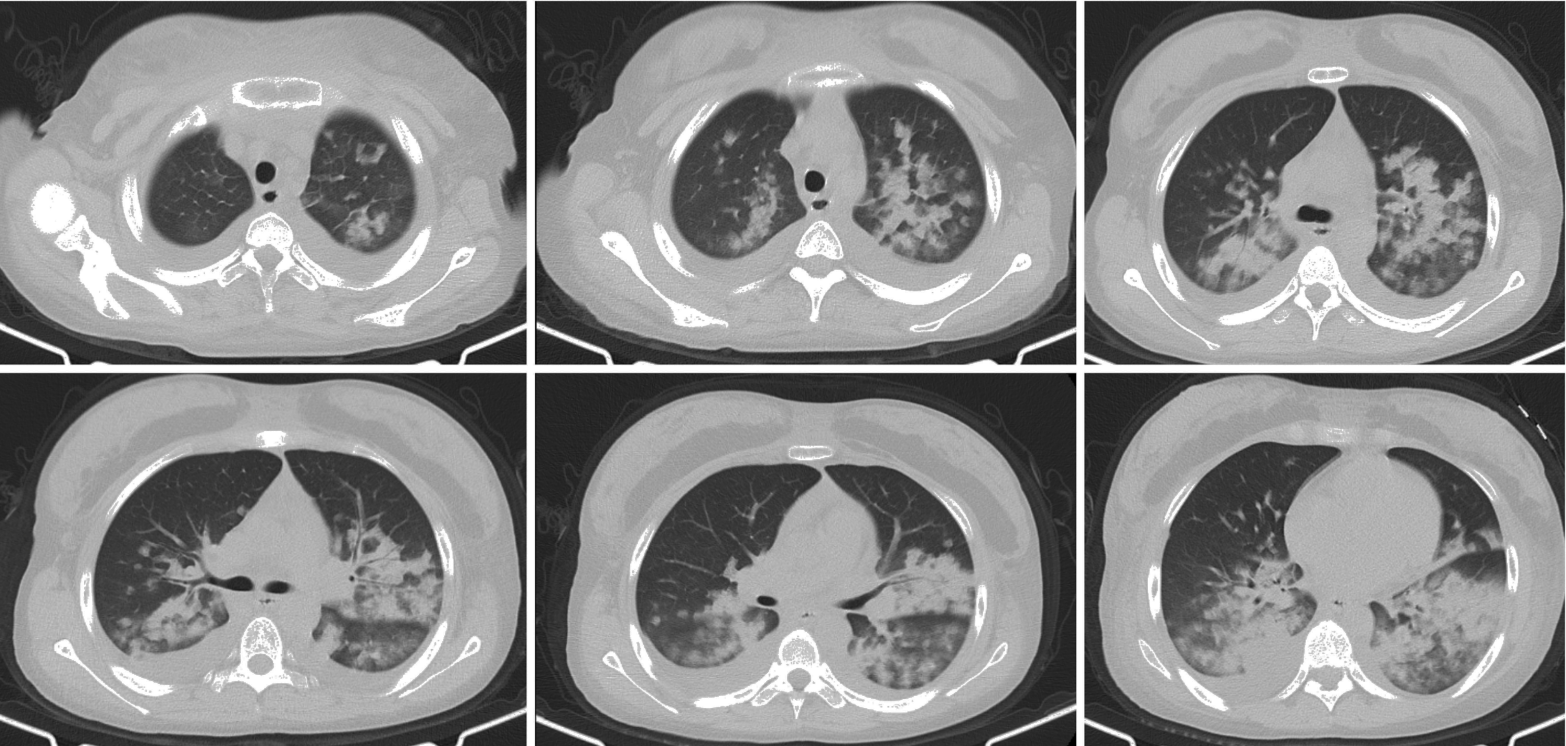
Chest computed tomography scan 1 day before admission. The lung window shows diffuse mixed ground-glass and consolidative opacities involving both lungs and small bilateral pleural effusion.

**Figure 2:**
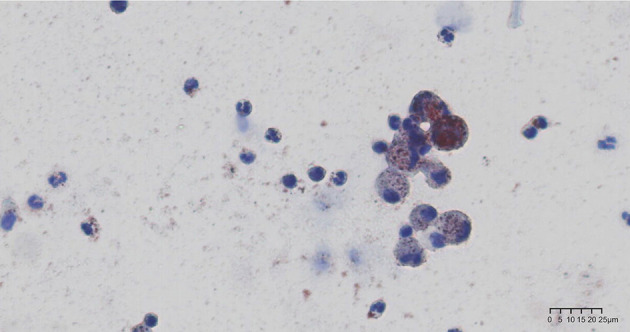
Oil red O staining of bronchoalveolar lavage fluid specimen. Alveolar macrophages with multiple prominent red- or brown-staining cytoplasmic inclusions seen under light microscopy.

Chest CT scan (Figure 1) revealed diffuse mixed ground-glass and consolidative opacities involving both lungs, thickening of the interlobular septa, bilateral pleural effusion, and subcutaneous soft tissue edema of the chest and back. CT pulmonary angiography (CTPA) showed no filling defect in the pulmonary artery. Echocardiography revealed an estimated pulmonary arterial systolic pressure of 36 mmHg, left ventricular ejection fraction of 70%, normal right ventricular systolic function, and normal inferior vena cava diameter. Brain CT showed no obvious abnormality. Given the suspicion of FES, bedside bronchoscopy performed within 24 h showed “mild inflammation”. Bronchoalveolar lavage was performed, and differential cell counts in the bronchoalveolar lavage fluid (BALF) sample showed macrophages, 10.8%; lymphocytes, 7.2%; and neutrophils, 82%. Cytopathologic evaluation of BALF revealed a multitude of tissue cells in the smear, with oil red O-positive bodies of different sizes and numbers in the cytoplasm of some cells, representing lipid droplets (Figure 2).

**Figure 3:**
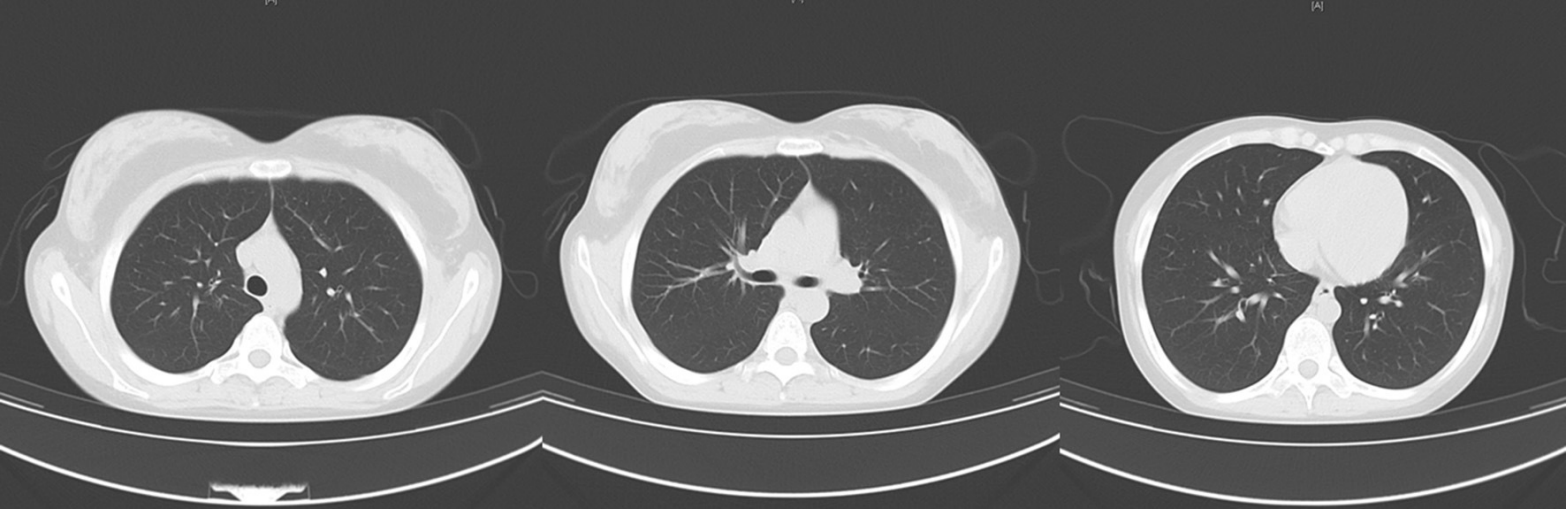
Chest computed tomography scan after discharge. There was no pulmonary infiltrates or pleural effusion.

Noninvasive ventilation was initiated. Methylprednisolone 80 mg daily and moxifloxacin 400 mg daily were given intravenously. Additionally, she received nutritional support and intravenous infusion of human albumin for 3 days. Her respiratory failure gradually improved. On day 5 of admission, her peripheral oxygen saturation was maintained >95% under air condition; subsequently, oxygen support was withdrawn, and methylprednisolone dosage was reduced to 40 mg daily for 2 days. She was discharged from the hospital on day 7 and was treated with prednisone acetate (40 mg qd followed by 20 mg qd for 5 days each). On reexamination 10 days after discharge, the patient's condition had improved. Her heart rate and peripheral SpO_2_ were 70–80 bpm and 98–99% on room air, respectively. Chest CT was normal (Figure 3), and prednisone was tapered to 10 mg daily for 3 days and then halted.

## Discussion and conclusions

Although the diagnosis of FES is challenging, researchers have tried to define diagnostic criteria. In 1974, Gurd and Wilson established the diagnostic criteria for FES (3), which are the most widely used in clinical practice, and Schonfeld developed a scoring scale for diagnosis in 1983 (4).

Our patient presented with acute hypoxemia, respiratory distress, and typical blizzard-like changes on chest CT hours after liposuction and a seriously low PaO_2_/FiO_2_ of 168, which were in accordance with acute respiratory distress syndrome (ARDS) diagnosis, graded as moderate based on Berlin Criteria of PaO^2^/FiO^2^ ratio 100–200 (5). The positive oil red O staining of BALF supported the diagnosis of pulmonary fat embolism; however, the patient did not show any skin or nervous system symptoms. Pulmonary fat embolism can lead to ARDS, which is one of the most serious complications and the main cause of death in such patients (6). ARDS is a kind of high permeability pulmonary edema, which is caused by many factors, and eventually results in diffuse alveolar injury (i.e., edema, inflammation, transparent membrane, atelectasis, or hemorrhage). Its clinical features are hypoxemia and double-lung infiltration shadows, accompanied by increased flow, increased physiological dead space, and decreased lung compliance.

Supportive care is the main therapy in the management of pulmonary fat embolism, with respiratory support being crucial. According to the severity of hypoxemia, the appropriate oxygen supply and respiratory support treatment should be determined to ensure oxygenation and minimize further lung damage. Corticosteroids are considered applicable and effective in cases of fulminant pulmonary fat embolism and ARDS, which can help reduce inflammation and fatty acid levels and improve oxygenation (2, 7). In a recent meta-analysis, the total mortality of FES was found to be 30.2%, and corticosteroid therapy was significantly associated with reduced mortality (7). There is a lack of enough evidence or recommendation about the appropriate dosage of corticosteroids. A low-dose regimen of glucocorticoids was administered in this case, in the form of methylprednisolone 80 mg daily for 5 days. Subsequently, the patient's respiratory failure was significantly relieved, and methylprednisolone was tapered. By contrast, the use of heparin is still controversial (1).

The most common CT features of pulmonary fat embolism are patchy ground-glass or nodular opacities, lobular septal thickening, or diffuse bilateral infiltrates consistent with ARDS (2, 8). The extent of ground-glass opacities and presence of consolidation correlates with disease severity (9). A retrospective review of radiological features on CTPA images of 15 patients diagnosed clinically with pulmonary fat embolism reported pulmonary opacity in 14 (93.3%) patients, ground-glass opacities in 9 (64.3%), alveolar opacities in 6 (42.9%), interlobular septal thickening in 10 (66.7%), and pleural effusions in 7 (46.7%) (9). Pulmonary artery filling defects were observed on CTPA only in 3 (20%) patients, suggesting that pulmonary artery filling defects are not commonly seen in patients with pulmonary fat embolism (10). In our case, the chest image showed diffuse exudation and consolidation in both lungs—considered to be a manifestation of ARDS secondary to pulmonary fat embolism—that was absorbed completely in 2 weeks, which is consistent with a previous report (10).

Another effective method for the diagnosis of pulmonary fat embolism is lung biopsy, although it is not the first choice because of its invasiveness. Round or oval negative staining observed in the blood vessels of the lung tissue section suggests potential fat emboli (1, 5). The lung tissue sections fixed in formalin can be stained with osmium tetraoxide solution and then embedded in paraffin. Round, uniform black droplets in the blood vessels indicate fat emboli. In addition, oil red O staining helps detect the presence of lipids in frozen sections and BALF, which appear red when visualized under light microscopy. The presence of lipid droplets in macrophages in BALF, especially if the number of cells positive for the staining is >30%, supports an FES diagnosis (11) and helps identify ARDS caused by other factors.

In the BALF pathological examination, routine processed pathological specimens (paraffin embedding, xylene dewaxing, and alcohol solvent dissolution) and cytospin specimens made from fresh cell suspension were examined separately in this case. Both specimens were stained with oil red O; the routine pathological specimens were negative for the staining, whereas the fresh cell slice was positive (Figure 2). During the processing of conventional pathological specimens, xylene and alcohol solvents lead to lipid dissolution, which may produce a false-negative result.

Pulmonary fat embolism is a serious complication after liposuction that can develop suddenly and progress rapidly. Early diagnosis and appropriate treatment are essential to reduce mortality and improve prognosis. Oil red O staining of BALF is helpful in the diagnosis of pulmonary fat embolism. Xylene and alcohol solvents should be avoided during the processing of specimens. Respiratory support and corticosteroid therapy are key in treating pulmonary fat embolism and ARDS.

## Data availability statement

The original contributions presented in the study are included in the article/supplementary material, further inquiries can be directed to the corresponding author.

## Ethics statement

The studies involving human participants were reviewed and approved by Peking University Third Hospital. The Ethics Committee waived the requirement of written informed consent for participation. Written informed consent was obtained from the patient for the publication of this manuscript and of potentially identifiable images or data included in this article.

## Author contributions

XG, XS, and QZ performed the study. XZ performed the histological examination and provided the photos. XG and QZ were major contributors in writing the manuscript. All authors analyzed and interpreted the patient data. All authors read and approved the final manuscript.
